# Breakfast Cereals Intended for Children: Opportunities for Reformulation and Potential Impact on Nutrient Intake

**DOI:** 10.3390/foods10081772

**Published:** 2021-07-30

**Authors:** Mariana Santos, Filipa Matias, Ana Isabel Rito, Isabel Castanheira, Duarte Torres, Isabel Loureiro, Ricardo Assunção

**Affiliations:** 1 Food and Nutrition Department, National Health Institute Dr Ricardo Jorge, Av Padre Cruz, 1649-016 Lisboa, Portugal; mariana.coelho@insa.min-saude.pt (M.S.); filipa.matias@insa.min-saude.pt (F.M.); ana.rito@insa.min-saude.pt (A.I.R.); isabel.castanheira@insa.min-saude.pt (I.C.); 2 NOVA National School of Public Health, NOVA University of Lisbon, Av Padre Cruz, 1600-560 Lisboa, Portugal; 3 Center for Studies and Research in Social Dynamics and Health (CEIDSS), Av Padre Cruz, 1649-016 Lisboa, Portugal; 4 Epidemiology Research Unit, Institute of Public Health, University of Porto, Rua Das Taipas 135, 4050-091 Porto, Portugal; dupamato@fcna.up.pt; 5 Faculty of Nutrition and Food Sciences, University of Porto, Rua Dr. Roberto Frias, 4200-465 Porto, Portugal; 6 NOVA National School of Public Health, Public Health Research Center (CISP), Comprehensive Health Research Center, NOVA University of Lisbon, Avenida Padre Cruz, 1600-560 Lisboa, Portugal; isalou@ensp.unl.pt; 7 CESAM, Centre for Environmental and Marine Studies, University of Aveiro, Campus Universitário de Santiago, 3810-193 Aveiro, Portugal

**Keywords:** breakfast cereals, nutrient profile, reformulation, food selection, children, health impact

## Abstract

Ready-to-eat cereals (RTECs) have become a popular breakfast option claiming to provide important nutrients to children’s diets, despite being a source of excess sugar and, therefore, a health concern. Thus, food reformulation constitutes an important public health strategy that could benefit from inputs provided by nutrient profiling. This study aimed to assess the adequacy of the RTECs for children available in Portuguese supermarkets, applying three nutrient profile models (NPMs)—the nutrient profile model of the World Health Organization’s Regional Office for Europe (WHO-EURO), the profile of the private-sector EU Pledge (EU-Pledge), and the national model developed by the Directorate-General of Health (NPM-PT)—in order to explore the potential for reformulation of the RTECs identified as not adequate and evaluate the impact of RTECs’ reformulation on the nutritional quality of Portuguese children’s diets. In total, 78 RTECs intended for children were assessed and two scenarios—current (not considering reformulation) and alternative (considering reformulation to accomplish the nutrient profile requirements)—were considered to assess the impact of reformulation on nutritional quality. Across all RTECs, only 5.1% could be promoted to children according to the considered NPMs. The most common nutrients requiring reformulation were sugar, saturated fatty acids (SFA), salt, and dietary fiber. The scenarios of reformulation considered could reduce the RTECs average content of total sugars, SFA, and salt by 43%, 8.7%, and 1.1%, respectively, and dietary fiber intake could be increased by 34%. Thus, these results support policies to implement reformulation strategies for developing healthier food products to be promoted to children.

## 1. Introduction

The benefits of breakfast, the first meal of the day, as part of a healthy and balanced lifestyle, are widely known from a nutritional, psychological, and societal point of view. The breakfast meal is related to several critical health factors that include healthy body weight, especially among children and adolescents [1]. The last “Health Behavior in School-aged Children” (HBSC) study, published in 2018 by the World Health Organization (WHO), revealed that the proportion of boys and girls eating breakfast on school days has declined since 2014, with just one out of two adolescents eating breakfast daily on school days. According to the same study, the proportion of Portuguese boys and girls who are 11 years old who eat breakfast on school days are 81% and 77%, respectively [2]. Among school children, ready-to-eat cereals (RTECs) seem to be one of the most popular components of the breakfast composition meal (66.5%) [3].

Breakfast cereals include several products grouped into processed cereals such as a porridge-type breakfast, and RTECs or “cold” breakfast cereals such as cornflakes and muesli [4]. In our study, we focused on RTECs such as flakes, puffed, and extruded cereals.

Several studies highlight that breakfast cereal consumption may be associated with improved cognitive functioning, with lower measures of overweight or obesity and reduced risk of hypertension [5].

RTECs consumption (compared with low/no consumption) is associated with higher nutrient intakes in the diets of European children and adolescents [6].

The consumption of RTECs has been associated with enhanced intakes of many vitamins and minerals in adults and children, either directly through its consumption or through its co-consumption with milk [7,8].

RTECs are high in carbohydrates and contain dietary fiber, proteins, vitamins, and minerals, which enables the recommended nutritional requirements of some nutrients to be met. Much of this effect is probably due to the fortification of many breakfast cereals [5].

RTECs may also contain high levels of salt and sugar, identified as risk factors for numerous chronic diseases when excessively consumed [9]. Observational and experimental studies indicated an association between high intake of added/free sugars and body weight [10], as well as with an increased risk factor for dental caries [11], and risk of developing several chronic diseases, namely type 2 diabetes [12] and cardiovascular diseases [13].

Initiatives are ongoing across the world to improve food environments and systems and to facilitate healthier children’s diets. Aligned with WHO recommendations, national governments across the globe are committed to developing efforts to limit the marketing of unhealthy foods to children [14,15].

The nutrient profile appears as one of the most relevant strategies to combine personal preference with a food healthy environment. Over the last years, nutrient profile models, which are tools to facilitate healthier food choice, have multiplied, and have been recognized by the industry and governments as a transparent and reproducible way of evaluating the healthfulness of foods. [16,17]. A large number of nutrient profile models have been developed covering different policy applications, highlighting those for regulating nutrition and health claims, marketing of foods for children, front-of-pack labelling (FOPL) systems, and food reformulation [18].

Focusing the restriction of food marketing and advertising to children, the WHO Regional Office for Europe (WHO-EURO) developed a nutrient profile model, and at the European Union (EU) level, several major international companies have signed the so-called EU-Pledge, as a collective self-regulation initiative establishing a code of conduct for marketing activities directed towards children [19,20].

At the national level, and in line with international recommendations, the Directorate -General of Health (DGS), developed a nutrient profile model (NPM-PT), adapting the WHO-EURO and adjusting the limits of some nutrients in certain categories of foods to align with the values defined by European Union legislation. Changes were also introduced to reflect the commitments established in Portugal regarding the food products’ reformulation [21].

The reformulation of food products becomes crucial to reduce the consumption of salt/sodium, sugar, and other nutrients relevant to public health and NP can be used as a tool to improve the overall nutritional quality of diets. On the other hand, reformulation should also consider ways to promote the consumption of positive nutrients like dietary fiber.

At the national level, the use of NPMs as tools to identify foods that can (or cannot) be marketed to children constitutes an emerging area of research in different countries. The purpose of this study is to add evidence on the validity of the NPM-PT, a NPM developed in Portugal, to correctly identify healthy foods, by comparing it with other models, as well as to test the capacity of the NPM-PT to guide a theoretical reformulation of RTECs marketed to children.

Particularly, the study aimed to answer the following questions: (i) what is the compliance rate of the RTECs for children available in the Portuguese supermarket, considering three nutrient profile models designed to restrict food marketing and advertising to children—the NPM-PT, WHO-EURO, and EU-Pledge; (ii) what is the potential for reformulation of the RTECs identified as not adequate; and (iii) what is the impact of RTECs reformulation on the nutritional quality of Portuguese children’s diets. Data from the present study intend to assist and support the industry companies, nutrition professionals, and policymakers to identify new avenues of research and regulatory actions associated with the implementation of reformulation strategies for developing healthier food products to be promoted to children. Especially at the European level, considering the European Single Market, there is a movement of goods, including foods. This means that any reformulation occurring in Portugal could have a reflection, and consequently, an impact, in other countries. Additionally, this contributes to promoting the harmonization of foods and nutrition-related policies across countries. At the national level, this research serves as a call for policymakers to consider the NPM-PT as a tool to promote the reformulation of RTECs and other child-targeted foods available in the market.

## 2. Materials and Methods

### 2.1. Breakfast Cereals Samples Selection

Overall, 78 children’s RTECs were selected from the available RTECs in the supermarkets from the Lisbon region. These products were chosen according to data from the Portuguese National Food, Nutrition, and Physical Activity Survey (IAN-AF) [22] for the consumption of RTECs by Portuguese children aged between 3 and 10 years old. The RTECs with gluten as a nutrition claim and organic labelling were also reported, according to data consumption from the Portuguese National Food, Nutrition, and Physical Activity Survey (IAN-AF). It was decided to also investigate these cereal categories (e.g., gluten-free (GF) and RTECs labelled organic for children), to assess their nutritional quality, as some studies reveal that products (RTECs) with a GF claim or labelled as organic are or are not nutritionally superior to regular products (RTECs) [23,24,25].

RTECs were identified when specifically remarked with ‘child’ or ‘children’ within the name or on the packaging; if the package enclosed puzzles or games directly geared toward children; if they presented direct claims or relevance fun or play; and if they were advertised for children on TV, films, or PC games [3,26,27]. The exclusion criteria for product selection were (i) incomplete images of the nutrition declaration or list of ingredients, (ii) unclear images on all sides of the package, and (iii) items that were marked as “product currently unavailable” on the website or in the supermarkets.

From each product, different information was collected, namely brand name, commercial product name, descriptive name, serving size, ingredient list, package marketing, and nutrition labelling information. Additionally, nutrition claims (presence or absence of gluten) [28] and organic certification information [29] were also registered. When needed, the data collection was also complemented through the extraction of information from corporate brand websites and online supermarkets. Data per portion size were obtained from packaging information or calculated based on the recommended serving size and nutrition labelling information about nutrient content; energy, total sugars, fat, saturated fatty acids, sodium, protein, and dietary fiber were also collected. Total sugars were considered as the amount of (total) sugars in prepacked foods to be listed, under carbohydrates in grams per 100 g, according to Regulation (EU) No 1169/2011 on the provision of food information to consumers. The contents of sodium per 100 g were estimated through the formula salt g per 100 g/2.5 [30].

RTECs were grouped according to the following categories as described by Goglia, R. et al. [31]—chocolate-flavored cereals, honey/caramel sweet cereals, filled cereals, and cornflakes/other plain cereals. The type of brand comprised two categories: national and store brand. Children RTECs without organic labelling were considered as conventional children RTECs. Some products such as plain oats, granola, and muesli with no added salt and/or sugar were excluded, as they did not fit into the defined categories (e.g., oat porridge). As the focus of the study was on product reformulation, the breakfast cereals sample selection only included RTECs with non-whole grain cereals (refined cereal flours).

All data collected (packaging information and their classification) were recorded on standardized spreadsheets usingMicrosoft Excel 2016 (Microsoft, Washington, WA, USA).

### 2.2. Nutritional Quality Assessment of RTECs Using Nutrient Profile Models

The marketing adequacy of the RTECs for children was assessed according to nutritional criteria established by three nutrient profile models, WHO-EURO [19], NPM-PT [21], and the most recent version of the EU-Pledge [20].

In the present study, the selected models reflect a category-based approach, supported by criteria for key nutrients. The compliance/non-compliance with the established criteria for the nutrients present per 100 g of food was evaluated, and each food product was classified as suitable/not suitable for promotion for children. These models were selected as they were developed to limit food marketing and publicity to children under 12–16 years, since they have a corresponding European/national scope. Characteristics of the three nutrient profile models used in this study are provided in Table A1.

### 2.3. Impact of Nutrient Profile Models on the Daily Nutrient Intake

RTECs that could be potentially reformulated were identified based on the NPM-PT criteria (presenting the most restrictive profile model), in combination with the dietary fiber criterion from the EU-Pledge, that considers a minimum value of 3 g of dietary fiber per 100 g of product. The dietary fiber criterion was included since the increased intake of fiber has been associated with the improvement of the overall quality of the diet among children and adults [32]. Furthermore, dietary fiber intake is usually below the dietary reference values, despite any improvement in its consumption, particularly in children, which is expected to be associated with health benefits [22,33].

For the consumption of RTECs by the Portuguese children aged between 3 and 10 years old, data from the Portuguese National Food, Nutrition, and Physical Activity Survey (IAN-AF) conducted from October 2015 to September 2016 were used [22]. Under the IAN-AF, the dietary intake was obtained via two non-consecutive one-day food diaries and with an additional personal interview with parents or other caregivers for more details related to the description and quantification of food [34]. Then, the intake of each food component was calculated and compared with the dietary reference values, established by the EFSA [35] and WHO [36]. Additionally, 20% of the daily energy and nutrient requirements for each age group was assumed as the reference intake for the breakfast meal [37]. Combining the nutrient contents and the consumption of these RTECs, the intake of each food component (total fat, saturated fatty acids, total sugars, salt/sodium, and fiber) was assessed. Nutrient contents corresponded to the amounts present in the product labels (for those products following the established criteria) or, alternatively, corresponding to the highest (salt, total sugar, total fat, and saturated fatty acids) or the lowest (fiber) amount established in the respective model (for those products that were not in accordance with the established criteria). Current (not considering reformulation) and alternative (considering reformulation to accomplish the nutrient profile requirements) scenarios were compared to assess the attainment of dietary recommended values (DRV) and, consequently, how these values could be impacted by a potential reformulation of RTECs.

### 2.4. Statistical Analysis

Once all RTECs information was collected, the reported 78 breakfast cereals were grouped into different categories regarding the ingredients (flavors), type of brand, and presence/absence of the gluten claim and organic labelling. This information was processed using different statistical methods. The Kolmogorov–Smirnov test was used to test the normality of the distribution of the variables to decide between parametric or nonparametric analyses for comparisons. Variables were expressed as the median (interquartile range).

The energy (kcal/100 g) and nutrient contents per 100 g of products were compared by the Mann–Whitney non-parametric test for two independent samples (for differences between the gluten and gluten-free categories and conventional and organic labelling categories, respectively) and using the Kruskal–Wallis non-parametric test with multiple pairwise comparisons (for differences among cereals categories). The Bonferroni correction was applied.

The level of convergence between the models was evaluated using the Cohen coefficient κ. The degree of agreement was scored as follows: 0.00–0.20 “light”, 0.21–0.40 “fair”, 0.41–0.60 “moderate”, 0.61–0.80 “substantial”, and 0.81–1.00 “almost perfect” [38,39].

Data were analyzed by using the Statistical Package for Social Sciences software (IBM SPSS Statistics, Version 26.0, IBM corp., Chicago, IL, USA). A result was considered statistically significant if the *p*-value was less than 0.05 and highly statistically significant for an observed *p*-value of less than 0.01.

## 3. Results

Table 1 summarizes the different categories of RTECs considered in the present study. Chocolate-flavored cereals (32, 41%) were the most represented, followed by the honey/caramel sweet cereals (19, 24%), cornflakes/other plain cereals (19, 24%), and filled cereals (8, 10%). Regarding the type of brand, the national brand (47, 60%) corresponded to the majority of RTECs considered, compared to the store brand (31, 40%). The presence of the gluten claim (68, 87%) and absence of organic labelling (conventional) (67, 86%) were represented the most.

Table 2 presents the observed variability in nutritional composition across all the categories of considered RTECs, concerning energy, total sugars, fats and saturated fatty acids, sodium, protein, and fiber.

Overall, the median energy value of RTECs was 385 kcal/100 g, but it varied widely among the cereal types (*p* = 0.0001). The energy content ranged from a median of 381 kcal/100 g for cornflakes/other plain cereals to 444 kcal/100 g for filled cereals.

The total sugars and total and saturated fatty acids contents diverged among the types of cereals (*p* < 0.0001), with the highest contents in filled cereals presenting 30.0, 15.0, and 4.6 g/100 g, respectively.

No statistically significant differences in protein (*p* = 0.445) and fiber (*p* = 0.341) contents were detected across the cereal types. Chocolate-flavored cereals presented the highest content of protein and fiber, at 7.6 and 5.1 g/100 g, respectively.

There were no statistically significant differences (*p* = 0.666) in the sodium content for the various types of cereals, with the chocolate-flavored cereals presenting the lowest content at 200 mg/100 g, and filled cereals presenting the highest content with 294 mg/100 g.

Regarding the remaining categories, comparing gluten-free products with gluten counterparts, the total sugars content showed statistically significant differences (*p* = 0.010). Overall, products carrying organic labelling presented lower total sugars at 4.2 g/100 g, total fat at 2.2 g/100 g, saturated fatty acids at 0.6 g/100 g, and sodium content at 8.0 mg/100 g. No statistically significant differences in fat (*p* = 0.066), protein (*p* = 0.235), and fiber (*p* = 0.818) contents were observed among the conventional children’s RTECs and children’s RTECs with organic labelling.

With the emphasis on the type of brand, the results revealed the highest values for energy, total sugars, and protein content for the store brand. The national brands presented the highest sodium content (280 mg/100 g). Statistically significant differences (*p* = 0.0004) within the type of brand in the total sugars content were found.

**Figure 1:**
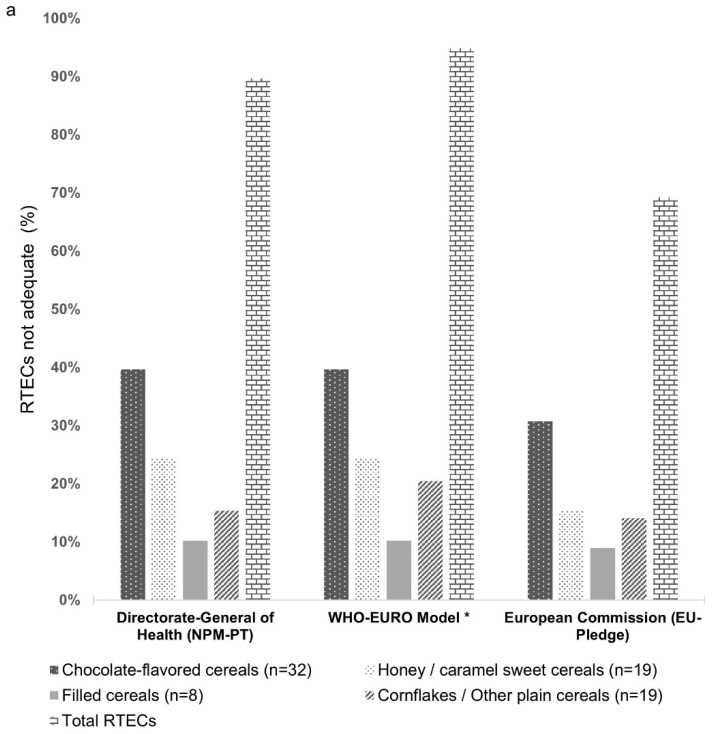

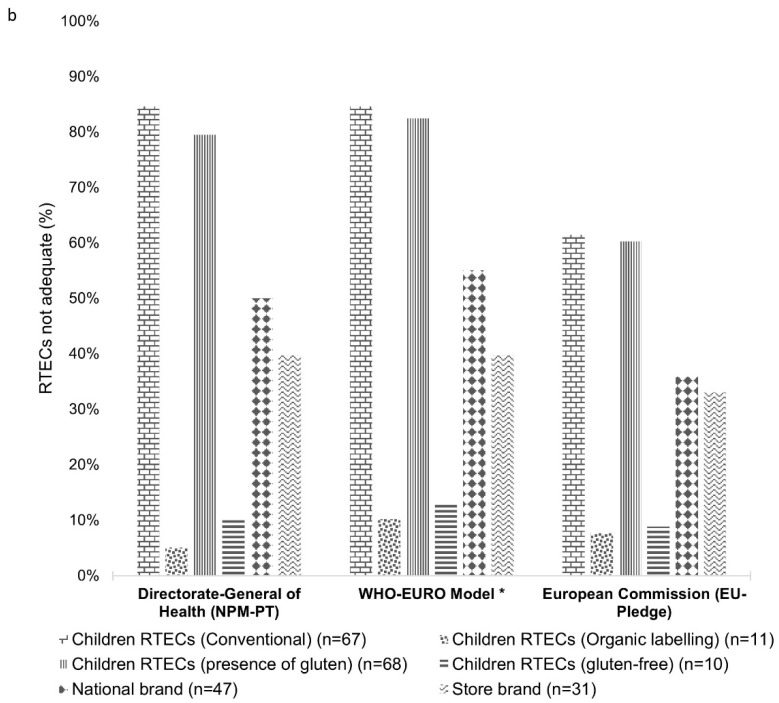
(**a**) Percentage of RTECs not adequate to be promoted by each nutrient profile model and (**b**) by nutrition claim (gluten), type of brand, and conventional/organic labelling. ^(^*^)^ For the WHO-EURO model application, the recommended minimum criterion fiber content of 6 g/100 g of product was included in evaluating the different RTECs.

Figure 1a,b presents the compliance of nutrient content of each RTECs compared to the criteria established in the three nutrient profile models, which considered the percentage of RTECs not adequate (%) to be promoted for children (in discordance with the criteria, at least for one nutrient).

Across all RTECs, the WHO-EURO model classified 95% (74) of the 78 cereals analyzed as non-adequate to be promoted for children. The corresponding figures for NPM-PT and EU-Pledge were 90% (70) and 69% (54), respectively, for all the products. The number of RTECs (*n* = 78) that met the three nutrient profiles developed to regulate food marketing to children was low; only 5.1% (*n* = 4) could be promoted to children according to the NPM-PT, WHO-EURO, and EU-Pledge models.

The conventional RTECs (*n* = 67) represent 86% of the cereals analyzed and the non-compliance rate was lower in the EU-Pledge model (*n* = 48, 62%) when compared to the NPM-PT and WHO-EURO models (*n* = 66, 85%).

The organic (*n* = 11) and gluten-free (*n* = 10) labelling of children’s RTECs represent 14% and 13% of the cereals analyzed, respectively, and gluten-free cereals showed a higher non-compliance rate (*n* = 8, 10%) compared with the organic RTECs (*n* = 4, 5.1%) for the NPM-PT. The EU-Pledge revealed a non-compliance rate similar for both categories of RTECs, 7.7% and 8.9%, respectively. The national RTECs brands (*n* = 47) represent 60% of the cereals analyzed and did not comply with the criteria of the NPM-PT (*n* = 39) and WHO-EURO (*n* = 43) in 50% and 55% of brands, respectively.

**Figure 2:**
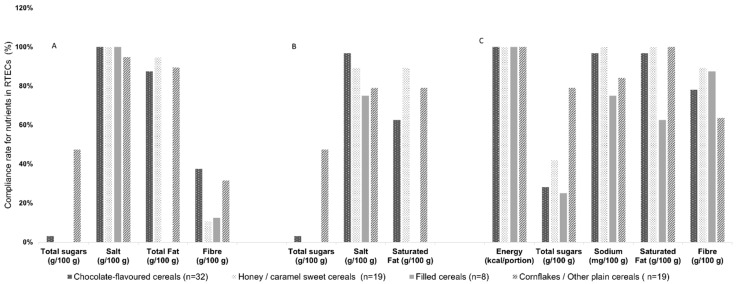
Percentage of agreement between RTECs’ nutrient content and the criteria set by the nutrient profile models: (**A**) the WHO-EURO model, (**B**) the Directorate-General of Health model (NPM-PT), and (**C**) the European Commission (EU-Pledge) model.

Figure 2 represents the compliance rate for the nutrient content of RTECs types with the criteria defined in the three nutrient profile models.

For total sugars, the WHO-EURO and NPM-PT models have the most restrictive criterion (<15 g/100 g), and consequently, none of the samples of filled cereals (*n* = 8) and honey/sweet caramel cereals (*n* = 19) complied with those models. Cornflakes/other plain cereals (*n* = 19) revealed a compliance rate of 47%. For the EU-Pledge model, the criterion is less restrictive for total sugars (<27 g/100 g) and the compliance rate ranged from 25% for filled cereals to 79% for cornflakes/other single cereals.

For sodium and salt, between 75% and 100% of the cereal types met the criterion for the three models under analysis.

The saturated fatty acids criterion has the most restrictive criterion in the NPM-PT model and the filled cereals type did not comply with the established criterion (≤1.5 g/100 g).

The recommended minimum fiber content of 6 g/100 g, is optional for the WHO-EURO model, and the chocolate-flavored cereals presented the highest percentage of products in this category (38%). For the EU-Pledge model, the minimum fiber content allowed is 3 g/100 g, with the honey/caramel cereals representing the highest percentage of products in this category (90%).

For energy criterion ≤210 kcal/portion (30 g), all types of RTECs complied with the criterion of the EU-Pledge model.

Analyzing the three different models, Table 3 indicates that substantial pairwise agreement was found between the WHO-EURO and the NPM-PT models. A fair agreement between the EU-Pledge and WHO-EURO models was identified. The NPM-PT model showed slight agreement with the EU-Pledge model.

These results allowed us to use the NPM-PT model as a reference for the reformulation of RTECs identified as not adequate, and two scenarios were considered—the current (not considering reformulation) and the alternative (considering reformulation to accomplish the nutrient profile requirements) scenarios. Table 4 summarizes the main results obtained regarding the estimated intake of the considered nutrients through the consumption of RTECs by the Portuguese children population, considering reformulation or no reformulation.

Analyzing the results of the estimations, with and without reformulation, total sugar and fiber are the nutrients where statistically significant differences were observed i.e., the decrease (in the case of total sugar) and increase (in the case of fiber) after reformulation are statistically significant when compared to the current scenario (without reformulation). Salt and SFA did not show statistically significant differences, which was already expected considering their usual contents in these types of products.

## 4. Discussion

Breakfast cereals are mentioned by several international surveys as having a high variability in their nutritional composition, in particular concerning sugar and salt content [40]. Simultaneously, robust results showed that consumption of RTECs, especially of fiber-rich or whole-grain RTECs, are implicated with several beneficial nutritional and health outcomes. Nevertheless, high total sugar intakes associated with frequent consumption of RTECs are a cause for concern [4].

Data from the present study do not (intentionally) include whole grains and ‘hot’ cereals, since this study focused on RTECs with non-whole grain cereals (refined cereal flours), where nutritional quality can be improved through product reformulation. This research aims to support industrial companies, nutrition professionals, and policymakers to identify actions associated with the implementation of reformulation strategies for the development of healthier food products to be promoted to children.

This study was one of the first in Portugal that addressed the nutritional composition and the adequacy of the RTECs for children available in the Portuguese supermarket, according to three nutrient profile models designed to restrict food marketing and advertising to children, including the model recently developed for Portugal.

According to the results obtained in the present study, the levels of total sugar in children’s RTECs are considerably higher, and 87% of RTECs did not comply with the sugar criterion (<15 g/100 g). Our findings are consistent with a previous study performed in Portugal, showing that 85% of children’s RTECs were non-compliant with the sugar criterion and 26% of children’s RTECs had more sugar than non-children’s (adult’s) RTECs [3].

The results of our study suggest that choosing organic children’s RTECs will result in a lower intake of total sugars, highlighted by our data, which are in line with the data from Germer et al.’s study [23]. A study published in 2018 focused on gluten-free products aimed at children and reported that gluten-free products contain lower amounts of sodium, total fat, saturated fatty acids, and sugar content, similar to the values of comparable products with gluten [41]. However, according to the present study, the total sugar content was lower and the total protein level was higher, when comparing gluten-free products with gluten counterparts.

Focusing on the type of brand, the higher values for total sugars observed in the in-store brand are in line with the results of the Ikonen et al. study. These authors concluded that despite being “tastier” and consequently presenting a higher consumer demand, these products are nutritionally poorer than other types of cereals [42]. The median fat (3.1 g/100 g) and saturated fatty acids contents observed in our study (1.1 g/100 g) were higher than the results indicated by Tong et al.’s study (1.1 g/100 g and 0.4 g/100 g, respectively) [43]. This discrepancy could be justified by the fact that, in the present study, filled cereals were considered, contrary to the Tong et al.’s study.

The sodium content of children’s RTECs found was 230 mg/100 g. Similar results were also reported by Tong et al., revealing a median content of 254 mg/100 g in children’s cereals [43]. Chepulis et al., in a cross-sectional analysis, described a median sodium content level of 240 mg/100 g in the UK for the children’s breakfast cereal with promotional characters [9].

Our data revealed, on average, that children’s breakfast cereals contained 4.9 g of fiber per 100 g. About 78% of ready-to-eat cereals are considered a “source of fiber” (≥3.0 g), but only 27% of ready-to-eat cereals are considered “high in fiber” (≥6.0 g) [28]. Assuming the child consumption of 30 g of breakfast cereals serving, they would ingest 1.5 g of fiber, which represents on average between 9% to 15% of the adequate intake. According to EFSA monitoring studies [35], the adequate intake of total fiber varies between 10 and 16 g/day for children between 3 to 10 years old. The consumption of RTECs represents an important source for dietary fiber intake from a single food category [44]. Fayet-Moore et al. reported that children who consume cereals at breakfast are more likely to meet their recommended intakes of B vitamins (niacin, thiamine, folate), calcium, iron, and fiber [45]. According to van den Boom et al., the consumption of RTECs is associated with a better nutritional profile in the diet of Spanish children, adolescents, and young adults. The breakfast has better quality in terms of food choices as well as energy and nutrient content [46].

Several studies suggest that children with diets consisting of higher amounts of fiber-rich foods generally consume less energy from total fat and saturated fatty acids and more dietary fiber in children’s diets might be associated with a lower risk of childhood obesity [32,47].

Comparing the different nutrient profile models used in the present study, we verified that the WHO-EURO is the most restrictive nutrient profile model followed by the NPM-PT. The EU-Pledge is the most tolerant regarding the restrictions to the marketing of foods for children. This was already described in different contexts [38,48].

Bonsmann et al. [48] observed that, from 2691 food products sold in the EU, 48% (EU-Pledge) and 68% (WHO-EURO) do not comply with the EU-Pledge and WHO-EURO nutrient profile criteria, respectively. Maschkowski et al. [24] also observed a lower compliance rate, with only 4–36% of German products meeting the criteria of the different nutrient profiles for cereals marketed to children.

Labonte et al.’s research revealed great variations in the degree of accuracy and agreement between nutrient profile (NP) models with applications in restricting the marketing of low nutritional quality food and beverages for children [49].

Recognizing the obtained results for the compliance of the RTECs available in Portugal with the nutrient profile models, the reformulation of RTECs and the associated impact of this reformulation on health constitutes an important aspect to be properly characterized.

The results from the Muth et al. study showed that reformulating a few categories of foods and beverages to reach the levels of products meeting an existing standard could improve dietary intake, particularly for saturated fatty acids, sugars, and fiber [50].

Trying to explore the potential for the reformulation of the RTECs identified as not adequate, the estimated intake of the nutrients considered in the NPM-PT model, as well as fiber, were estimated. Analyzing the results of the simulations with and without reformulation, we observed a reduction of the total sugar intake from 17 g/day to 10 g/day, representing a reduction of 43% for the total sugar intake from RTECs. Combet et al., who tested the capacity of a multi-nutrient profiling system for product reformulation, found average reductions between 19% and 38% [51].

Food reformulation can be a challenge, especially as nutrients such as sodium, fat, and sugar often play a role in the technological and sensorial functions in these products. Sugar, for example, is used for taste or texture, for preservation, and as a bulking agent, and salt is a preservative that prevents spoilage [52,53].

In Portugal, food products’ reformulation was established under co-regulation agreements with the food industry in the framework of the Integrated Strategy for the Promotion of Healthy Eating (EIPAS) [54]. The “Food Industry Co-Regulation Agreement” covers the reformulation of several food products high in salt, sugar, and trans fatty acids, as well as the main dietary sources of these nutrients, for the Portuguese population. The principles of the reformulation include reducing salt content between 12% and 30% and sugar content between 7% and 10% by 2021. The current agreement covers the reformulation of breakfast cereals establishing a reduction target of 10% for salt (sodium) and sugar contents. This will be achieved by setting progressive reduction targets for each food and drink category [55].

Food reformulation strategies based on setting targets for progressive reduction or increase of key nutrients are crucial to avoid consumers switching to another product or changing the amount they consume when, for example, the product’s levels of added sugar or salt are changed. The taste and texture of foods after reformulation will also influence consumption at a population level [56,57,58].

Considering the WHO recommendations for the intake of free sugars per day (<5% Energy) [36], children of 3 to 10 years old should have an upper daily limit of free sugars of 14–23 g [35], respectively. In this study, if we assign 100% of total sugars as added sugar for RTECs [59], and consider that the breakfast meal represents 20% of the energy of children’s daily reference values and nutrient intake, the reformulation scenario showed the RTECs representing 44–73% of the daily reference value for free sugars through the consumption of RTECs. The reformulation scenario contributes to a considerable reduction in total daily sugar intake from RTEC, although these still represent a higher contribution to the daily free sugars intake, in particular for young children.

For most lipid components, no reference values are proposed by EFSA [35]. In this case, we decided to follow the nutrient-based standards for planning nutritionally balanced menus developed by Public Health England, which recommend not more than 11% of energy as a reference value for saturated fatty acids [37]. For a 1500 kcal diet, this would be 18 g SFA per day. The reformulation scenario showed that RTECs contributed to 3.3% of the total SFA intake, reflecting a non-significant result to the contribution to SFA intake and diet quality.

On the other hand, for salt intake, children of 3 to 10 years old should have an upper daily limit of salt of 2.75–4.25 g [35], respectively. Our study revealed that salt intake through breakfast cereals consumption ranged between 0.444 g/day for the scenario without reformulation (current formulation of breakfast cereals) to 0.439 g/day for the scenario with reformulation, which represents a reduction of 1.1% in daily salt intake. These results showed that cereals have been produced with lower levels of salt across all of the different cereal categories considered, and this represents a non-significant contribution to salt intake and diet quality of the RTECs. Pombo-Rodrigues et al.’s study also described similar results concerning the sodium or salt levels in RTECs [60].

According to EFSA, the adequate intake for fiber is 10–16 g/day for children between 3 to 10 years old [35]. Considering the reformulation of RTECs, we observed an average increase of 34% in the daily dietary fiber intake. IAN-AF revealed that the mean intake of fiber was 14 g per day for children under 10 years. The present results support the inclusion of dietary fiber in the NPM-PT model, contributing to the improvement of its intake and complying with the recommendations for fiber dietary recommendation intake.

Comparing two typical Portuguese children’s breakfast options of RTEC children consumers (250 mL of milk + 30 g portion of RTECs reformulated + 50 g portion of bread) and non-RTEC children consumers (250 mL of milk + 50 g portion of bread) at breakfast, the RTEC consumers have a nutrient intake on average of 23.5 g total sugars, 1.4 g salt, 3.1 g saturated fat, and 4.6 g fiber, representing total sugars as 6.0% of the total energy intake (TEI), salt between 33% and 51%; fiber between 33% and 46%, and saturated fat at 1.9% of the TEI of the daily nutrient intake in children. For non-RTEC children consumers, the breakfast provides a nutrient intake on average of 13.5 g total sugars, 0.97 g salt, 2.4 g SFA, and 2.2 g fiber, representing total sugars with 3.4% of the TEI, salt between 23% and 35%; fiber between 16% and 22%, and saturated fat with 1.4% of the TEI of the daily nutrient intake in children [3,35,37,61]. Compared with non-cereal breakfast consumers, breakfast cereal consumers had higher intakes of dietary fiber and total sugars. This finding is consistent with Fayet-Moore et al.’s study [45].

These results can provide the food industry with an overall picture of RTECs’ nutrient daily intake contribution to modify the products to be healthier and more beneficial.

This is the first study applying a national policymaking tool, the NPM-PT model, to classify RTECs as suitable/unsuitable for marketing to children. In this study we assessed the potential of nutrient profiling models to guide food reformulation, contributing to changing the food environment and improving children’s nutritional intake. In order to reach its full potential, reformulation should be implemented across the food sector.

Further studies are needed to suppress some drawbacks of this study including the following:

- For some categories of cereals (e.g., gluten-free and organic children’s RTECs) the number of samples considered was low (<15); however, the sample reflected the products available in the market.
- The presented results constitute a snapshot of the RTECs available on the market in a particular period.
- The reformulation scenario is a theoretical study; therefore, we did not assess the sensory properties of RTECs after reformulation, and we assumed that children will consume the same amount of reformulated RTECs. Future research should address the issues raised here, including sensory analysis and determining the children’s acceptability of these new products.
- This research reflected only RTECs, and upcoming research focusing on other food groups, e.g., fruit-based snacks, bread and bakery products, convenience foods, and yogurts, will allow for a more accurate nutritional profile to be defined for food products marketed to children.
- Access to data on added sugars would help to identify the exact amount of sugar consumed that was added to food during its production.
- Easier access to composition data of high-quality branded food is also needed.

Finally, future research should explore the impact of reformulation of target nutrients on health outcomes and quality of life measures i.e., quality-adjusted life years (QALYs) and disability-adjusted life years (DALYs).

## 5. Conclusions

Although nutrient profiling does not address all aspects of nutrition, diet, or health, it is a helpful tool to use in conjunction with interventions aimed at improving diets. It can also be used in implementing the recommendations on the marketing of foods to children.

From our study, 87% of RTECs had a sugar content >15 g/100 g, responsible for the highest percentage of non-compliance in the evaluation by the various nutrient profile models.

Considering the used models, NPM-PT provides very similar results to the WHO nutrient profile model in terms of its purpose, enabling a good level of agreement.

As demonstrated in the current study, the process of reformulation of RTECs, namely, to reduce salt and sugar contents, is recommend and should not compromise their global nutritional profile. It is important to define feasible reduction targets without having to replace them with other ingredients, such as sugar by sweeteners. Additionally, it is necessary to define a gradual reduction to achieve the adaptation by the consumer to products with a lower amount of salt and sugar.

To summarize, this study provides the food industry with a general picture of children RTECs’ nutritional profiles, mainly focusing on reducing sugars and salt and increasing fiber, offering healthier products.

## Figures and Tables

**Table 1:** RTECs samples distribution per category according to common characteristics.

Type of Cereals	*n*	%
Chocolate-flavored cereals	32	41
Honey/caramel sweet cereals	19	24
Filled cereals	8	10
Cornflakes/other plain cereals	19	24
Organic/conventional		
Children RTECs (conventional)	67	86
Children RTECs (organic labelling)	11	14
Nutrition claim (gluten)		
Children RTECs (presence of gluten)	68	87
Children RTECs (gluten-free)	10	13
Type of brands		
National brand	47	60
Store brand	31	40

**Table 2:** Energy, macronutrients, and sodium across the different children RTECs categories considered in the study.

	Categories		Energy kcal/100 g	Total Sugars (g/100 g)	Fat (g/100 g)	Saturated Fatty Acids (g/100 g)	Sodium (mg/100 g)	Protein (g/100 g)	Fiber (g/100 g)
Category	Children’s RTECs (*n* = 78)	median (25th–75th percentile)	385 (380–402)	28.3 (24.0–31.0)	3.1 (2.0–7.2)	1.1 (0.6–2.0)	230 (123–312)	7.3 (6.0–8.4)	4.6 (3.0–6.0)
		SD	26	9.5	4.8	1.6	155	1.6	2.8
		*p*-value	0.002	0.003	0.029	0.01	0.040	0.584	0.43
Type	Chocolate-flavored cereals (*n* = 32)	median (25th–75th percentile)	383 (378–394)	29.0 (27.0–32.0)	2.7 (2.5–4.5)	1.3 (1.0–2.0)	200 (161–276)	7.6 (6.5–8.9)	5.1 (3.5–6.5)
		SD	24	4.7	3.9	1.2	101	1.2	1.9
	Honey/caramel sweet cereals (*n* = 19)	median (25th–75th percentile)	388 (380–399)	28.0 (24.1–33.0)	3.0 (1.50–5.0)	0.6 (0.3–1.3)	280 (12.0–392)	6.9 (6.0–8.3)	4.5 (3.2–5.5)
		SD	13	7.9	2.8	0.6	166	1.3	1.7
	Filled cereals (*n* = 8)	Median (25th–75th percentile)	444 (440–449)	30.0 (27.1–32.7)	15.0 (14.2–15.9)	4.6 (3.9–6.4)	294 (135–414)	7.5 (7.0–8.5)	3.7 (3.0–4.2)
		SD	13	3.3	2.3	1.5	155	1.1	1.1
	Cornflakes/other plain cereals (*n* = 19)	Median (25th–75th percentile)	381 (379–385)	22.3 (1.1–27.0)	2.2 (1.4–7.1)	0.6 (0.3–1.3)	260 (12.0–400)	7.0 (6.0–8.4)	3.8 (2.2–6.6)
		SD	19	11.9	3.6	1.0	217	2.4	4.8
		*p*-value	0.000120	0.000113	0.000053	0.00000017	0.666	0.445	0.341
Nutrition claim (gluten)	Children’s RTECs (presence of gluten) *n* = 68	Median (25th–75th percentile)	386 (380–408)	28.9 (24.7–31.8)	3.2 (2.1–7.4	1.0 (0.6–2.3)	230 (125–312)	7.3 (6.5–8.5)	4.7 (3.0–6.0)
		SD	28	9.3	5.0	1.7	140	1.6	2.0
	Children’s RTECs (gluten-free) *n* = 10	Median (25th–75th percentile)	382 (379–385)	21.7 (11.3–28.4)	2.5 (1.4–7.3)	1.3 (0.6–1.7)	228 (21.0–363)	7.5 (6.2–8.4)	4.1 (3.2–9.8)
		SD	5	9.1	3.5	0.6	244	1.3	5.7
		*p*-value	0.173	0.010	0.402	0.934	0.994	0.905	0.828
Organic/ conventional	Children’s RTECs conventional *n* = 67	Median (25th–75th percentile)	388 (380–409)	28.8 (24.9–31.0)	3.2 (2.2–9.3)	1.2 (0.6–2.3)	240 (140–312)	7.3 (6.5–8.4)	4.7 (3.2–6.0)
		SD	26	6.1	5.0	1.7	148	1.1	2.7
	Children’s RTECs organic *n* = 11	Median (25th–75th percentile)	378 (357–384)	4.2 (1.0–24.0)	2.2 (1.4–3.6)	0.6 (0.3–0.7)	8.0 (4.0–256)	7.6 (6.6–11.0)	3.6 (2.6–9.0)
		SD	16	14.2	1.8	0.4	148.2	2.9	3.3
		*p*-value	0.003	0.000254	0.066	0.002	0.003	0.235	0.818
National/ Store/	Children’s RTECs national *n* = 47	Median (25th–75th percentile)	384 (378–402)	25.0 (21.0–29.0)	3.2 (2.1–7.6)	1.1 (0.6–2.0)	280 (80.0–392)	7.2 (6.0–8.1)	4.6 (3.0–6.2)
		SD	26	10.5	4.6	1.3	177	1.8	3.3
	Children’s RTECs store brand *n* = 31	Median (25th–75th percentile)	390 (380–406)	30.0 (27.0–32.9)	2.6 (2.0–5.0)	1.1 (0.7–2.3)	200 (140–280)	7.5 (6.6–9.0)	4.5 (3.0–5.6)
		SD	27	5.3	5.2	1.9	112	1.3	1.7
		*p*-value	0.158	0.000446	0.721	0.487	0.254	0.240	0.646

Values are expressed as median (25th–75th percentile); SD = standard deviation; *p*-value obtained with Kruskal–Wallis tests for independent samples with multiple pairwise comparisons (breakfast cereals categories) or with the Mann–Whitney test for two independent samples (gluten/gluten-free; conventional/organic labelling; national/store brand).

**Table 3:** Pairwise k values calculated for the three models considered.

	WHO-EURO	NPM-PT
NPM-PT	0.642 ***	-
EU-Pledge	0.217 **	0.114 *

*, slight; ** fair; *** substantial agreement.

**Table 4:** Estimated intake of total sugar, salt, saturated fatty acids (SFA), and dietary fiber through the consumption of RTECs by the Portuguese children population, considering reformulation (estimates considering the children consumption).

	Without Reformulation	With Reformulation ^a^
	Total Sugar (g/day)	
Mean	17.5 ± 0.7 **	10.0 ± 0.4 **
Median	15.8	9.0
Minimum	0.2	0.2
Maximum	83.2	48.0
	Salt (g/day)	
Mean	0.444 ± 0.019	0.439 ± 0.019
Median	0.408	0.408
Minimum	0.002	0.002
Maximum	2.720	2.720
	SFA (g/day)	
Mean	0.654 ± 0.032	0.597 ± 0.027
Median	0.495	0.458
Minimum	0.018	0.018
Maximum	2.94	2.64
	Fiber (g/day)	
Mean	1.83 ± 0.09 **	2.46 ± 0.1 **
Median	1.38	1.98
Minimum	0.09	0.13
Maximum	8.59	9.60

** *p* < 0.001 for the statistical comparison of RTECs intake, without and with reformulation; ^a^ Reformulation scenario performed according to the NPM-PT (for total sugar, salt, and SFA) and EU-Pledge (for fiber).

## Data Availability

The data presented in this study are available on request from the corresponding author.

## Appendix A

**Table A1:** Characteristics of the three nutrient profile models considered in this study.

Models	Purpose	Food Categories	Type of Model	Classification Criteria	Breakfast Cereal Category		
					Nutrients to Limit	Nutrients to Encourage	Nutritional Criteria
Directorate-General of Health (NPM-PT) model [21]	Defining better options in the context of marketing food and beverage to children ≥36 months and <16 years.	20	Threshold	Food category specific	Total sugars, salt, and saturated fatty acids	none	Total sugars: ≤ 15 g/100 g ^(1),(2)^ Salt: ≤1.0 g/100 g ^(3)^ Saturated fatty acids: ≤1.5 g/100 g ^(4)^
WHO-EURO model [19]	Defining better options in the context of marketing food and beverage to children ≥36 months and <16 years.	17	Threshold	Food category specific	Total sugars, salt, and total fat	Fiber ^(5)^	Total sugars: ≤15 g/100 g ^(1), (2)^ Salt: ≤1.6 g/100 g Total fat: ≤10 g/100 g Fiber: >6 g/100 g ^(5)^
European Commission (EU-Pledge) model [20]	Defining better options in the context of advertising food and beverage to European children under the age of 12 years old on TV, print, and the internet.	9	Threshold	Food category specific	Energy, total sugars, sodium, and saturated fatty acids.	Fiber	Energy: ≤210 kcal/portion (30 g) ^(6)^ Total sugars: ≤27 g/100 g ^(7)^ Sodium: ≤450 mg/100 g Saturated fatty acids: ≤5 g/100 g Fiber: ≥3 g/100 g

^(1)^ The WHO-EURO and the NPM-PT models consider the same criterion for total sugars (<15 g/100 g), representing approximately 25% of the reference intake per 100 g needed to be identified as “high sugar content” product [21]. ^(2)^ Total sugars refers to the total sugar content of the food product, which may be composed of: intrinsic sugars incorporated within the structure of intact fruit and vegetables; sugars from milk (lactose and galactose); and all additional monosaccharides and disaccharides added to foods by the manufacturer, cook, or consumer, plus sugars naturally present in honey, syrups, and fruit juices [19]. ^(3)^ For the salt content, the criterion of 1 g (instead of 1.6 g) was established, as this was the target set in the agreement with the food industry and distribution for the reformulation of this food category [21]. ^(4)^ For the category breakfast cereals, considering the impossibility to set a value for the total fat content, the limit for the content of saturated fatty acids was added according to the value of the claim “low saturated fat”—1.5 g, as defined in Regulation (EC) No 1924/2006 [28]. ^(5)^ For this category, countries may choose to include a limit for the minimum fiber; the WHO proposes the minimum value of >6 g dietary fiber. ^(6)^ The reference unit for the energy is established per portion (kcal/portion). For breakfast cereals, the portion ranges from 30 g to 45 g, although in our study, only the results for 30 g were considered. ^(7)^ For total sugars, the considered criterion was 27 g/100 g, a value that corresponds to <11% of the children’s reference value (based on 85 g sugar/day).
